# Elucidating the role of stress signalling in ALS

**DOI:** 10.1186/1753-6561-9-S1-A15

**Published:** 2015-01-14

**Authors:** N Nurdin, M Hogg, J Prehn

**Affiliations:** 1 Department of Physiology, 123 St. Stephen’s Green, Royal College of Surgeons in Ireland, Dublin, Ireland

## Background

ALS is a fatal, rapidly progressive neurodegenerative disorder affecting motor neurons in the CNS; this results in muscle weakness which progresses to paralysis and death from respiratory failure. There is currently no effective cure as its pathophysiology is poorly understood; however, aggregates comprising misfolded proteins are known to be characteristic of the disease. These protein aggregations could elicit ER stress and subsequently the unfolded protein response (UPR). Initially, this response is cytoprotective as it inhibits protein synthesis thereby preventing further protein accumulation until the stress resolves, however if prolonged it can stimulate apoptosis. This study attempts to clarify the role of ER stress and the UPR in ALS.

## Methods

Immunofluorescence was performed on SOD1 mice spinal cord sections at two time points, PND90 and endstage, which were compared to wild type controls. Antibodies were used against ER stress markers ATF6, CHOP, PERK, IRE1 and p-eif2a with SMI32, NeuN or GFAP used as co-stains for distinguishing the cell type and signal co-localization. Immunofluorescence was then optimized to eliminate autofluorescence and antigen masking in the FFPE human tissue. Human spinal cord samples from 5 patients with ALS and 5 controls were then analysed by Nissl staining to assess the histology and with the ATF6 antibody to assess levels of ER stress.

## Results

P-eif2a, nuclear ATF6, CHOP and PERK levels were elevated in the endstage transgenic mice compared to the PND90 and wild type samples. The p-eif2a co-localised with the NeuN neuron stain. Further results from the human tissue immunofluorescence are pending.

## Conclusions

ER stress was shown to be increased in the SOD1 mouse model compared to controls; these mice are a model for ALS as 20% familial ALS cases carry the same mutation. Co-localisation of the NeuN with the p-eif2a implies a neuronal location of the stress. This indicates the UPR’s involvement in the pathophysiology of ALS and suggests it may be a delayed response or a consequence of the disease as markers were exceedingly elevated in the endstage compared to the day 90 mouse spinal cords. The ER stress pathway could potentially be a novel therapeutic target in ALS with many emerging drugs modifying the UPR and if identified to be raised in other more accessible tissue in ALS patients it could potentially be used as a biomarker for early disease diagnosis.

